# Disease Risk Assessment Using a Voronoi-Based Network Analysis of Genes and Variants Scores

**DOI:** 10.3389/fgene.2017.00029

**Published:** 2017-03-07

**Authors:** Lin Chen, Gouri Mukerjee, Ruslan Dorfman, Seyed M. Moghadas

**Affiliations:** ^1^Agent-Based Modelling Laboratory, York UniversityToronto, ON, Canada; ^2^GeneYouIn Inc.Maple, ON, Canada

**Keywords:** Voronoi tessellation, cluster analysis, disease risk assessment, gene-variant scores, data analysis

## Abstract

Much effort has been devoted to assess disease risk based on large-scale protein-protein network and genotype-phenotype associations. However, the challenge of risk prediction for complex diseases remains unaddressed. Here, we propose a framework to quantify the risk based on a Voronoi tessellation network analysis, taking into account the disease association scores of both genes and variants. By integrating ClinVar, SNPnexus, and DISEASES databases, we introduce a gene-variant map that is based on the pairwise disease-associated gene-variant scores. This map is clustered using Voronoi tessellation and network analysis with a threshold obtained from fitting the background Voronoi cell density distribution. We define the relative risk of disease that is inferred from the scores of the data points within the related clusters on the gene-variant map. We identify autoimmune-associated clusters that may interact at the system-level. The proposed framework can be used to determine the clusters that are specific to a subtype or contribute to multiple subtypes of complex diseases.

## Introduction

Rapid advances in exome sequencing technology combined with the development of novel genomic annotation approaches in the last two decades have provided important vistas for assessing the risk of complex disorders (McCarthy et al., 2008; Majewski and Pastinen, 2011). Understanding the genotype-phenotype interactions *via* genome-wide association studies (GWAS) and large-scale protein-protein network and functional pathways, has been the focus of much research in risk assessment (Leiserson et al., 2013; van der Sijde et al., 2014). Complex diseases, however, are tightly associated with rare variants, often with a low minor allele frequency but high-penetrance (Fearnhead et al., 2004, 2005; Bodmer and Bonilla, 2008; Manolio et al., 2009), which challenges the current GWAS, focusing on common variants (Satake et al., 2009; Jia et al., 2010). Understanding the combinatory effects of variants on complex disorders and the interactions between them are critical in disease risk modeling (Okser et al., 2013). Therefore, the inclusion of both common and rare variants in the network, based on their association with the disease, is essential to modeling and quantifying the risk of complex disorders.

Several network and systems biology approaches have recently been developed to infer the risk of disease based on the integration of genome-wide expression data (Parikshak et al., 2015), identification of disease-causative variants *via* large-scale genome-wide analysis (Krawczyk et al., 2010; Gratten et al., 2014), mapping protein-protein interaction information (Rual et al., 2005; Wang et al., 2012), statistical inference on the connectivity between molecular nodes (Goh et al., 2007; Gilman et al., 2011, 2012; Chang et al., 2015), and functional annotation using pathway databases (Parikshak et al., 2013). Such databases include Kyoto Encyclopaedia of Genes and Genome Elements (KEGG) (Ogata et al., 1999; Kanehisa and Goto, 2000) and Gene Ontology (GO) (Ashburner et al., 2000). However, these approaches mainly deal with network analysis at the gene level without specifically considering the disease-associated scores of variants in clustering or risk assessment.

On the gene level, complex diseases are affected by various perturbations in the genetic architecture that integrates the functional pathways and interactions between genes (Carter et al., 2013). Substantial efforts have been made to identify critical genes clusters as the potential causes of disease development. These include predicting protein-protein interaction (PPI) network and subnetworks using Markov cluster algorithm (Rual et al., 2005; Stelzl et al., 2005; Sun et al., 2011), or using background network based on the likelihood of genetic interactions and phenotype association to identify functional clusters associated with disease-related *de novo* CNVs (Gilman et al., 2011, 2012). However, integrative approaches that incorporate the contribution of both genes and variants to infer disease risk quantitatively are still lacking.

In an attempt to develop such an approach, we integrated databases of disease-associated genes and variants scores and applied the well-established method of Voronoi tessellation in the Euclidean coordinate for clustering and network analysis (Ebeling and Wiedenmann, 1993; Ramella et al., 2001; Edla and Jana, 2011). Given a set of data points, a Voronoi diagram is a partition of the space into cells, where a cell corresponding to a given data point is a locus of all points of space closest to this data point. Voronoi tessellation is commonly used in various fields of natural and medical sciences (Okabe et al., 1992, 2000; Aurenhammer, 1993; Ebeling and Wiedenmann, 1993; Ramella et al., 2001; Dupanloup et al., 2002; Wieland et al., 2007; Bishnu and Bhattacherjee, 2009; Kao et al., 2010; Edla and Jana, 2011), and in geographic information systems to define the partition cell, or catchment areas containing individual sites by their influence (Okabe et al., 1992, 2000).

In recent years, there has been a surge of interest in using Voronoi-based clustering for biological data. For instance, Edla et al. (Edla and Jana, 2011) presented Voronoi clustering algorithms that filtered the Voronoi neighbors of biological data points based on the distance between neighbors. Bishnu (Bishnu and Bhattacherjee, 2009) used Voronoi tessellation to cluster centroids following initial clustering *via K*-means method. Ramella et al. (Ebeling and Wiedenmann, 1993; Ramella et al., 2001) determined the threshold of clustering for biological data by using Kiang distribution fitted to the background Voronoi cell density distribution. Building on the previous literature, in this study, we propose a framework to quantitatively infer disease risk based on the clusters identified by Voronoi tessellation and network analysis of a score-based gene-variant map.

## Materials and methods

To develop our quantitative method, we parsed distinct databases and integrated the information necessary for clustering based on genes and variants scores. To identify the clusters, we applied Voronoi tessellation network analyses, which have been widely used for data aggregation and clustering (Wieland et al., 2007; Balcan et al., 2009). Here we propose the relative risk using a Voronoi network algorithm that identifies disease-associated clusters containing genes, whose Voronoi cell densities were above a certain threshold, obtained from the Chi-square distribution fitted to the background data.

### Integration of databases on genes and variants

**Figure 1:**
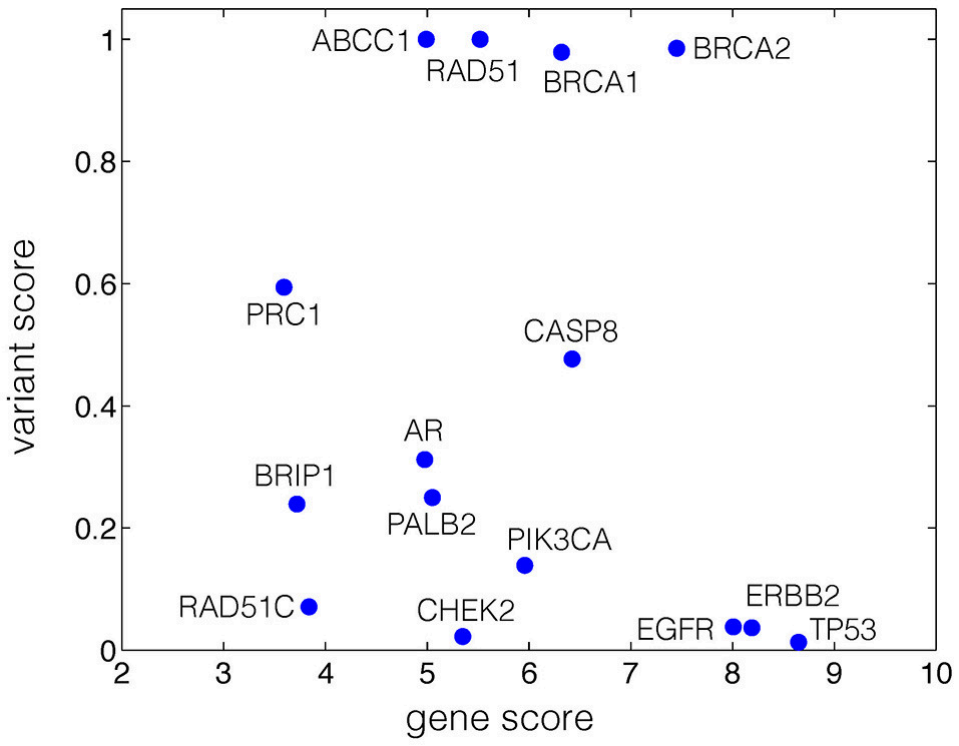
**Gene-variant map of breast cancer, generated based on the disease association scores at both gene and variant levels**. The x-axis represents the Z-score of genes with breast cancer association. The y-axis represents the ratio of 1-SIFT scores of variants associated with breast cancer to that of all variants for each gene.

The disease associated information of variants was obtained from the integration of various databases. These databases contained information on the clinical relevance of the variants from ClinVarFullRelease_00-latest.xml file in ClinVar^1^,^2^, and the functional scores of the variants from SNPnexus (Chelala et al., 2009; Dayem et al., 2012, 2013). The xml file from ClinVar was parsed *via* customized scripts using ElementTree in open source software Python^3^. The phenotypes for each variant were obtained from “Trait/Name” under “TraitSet” from the ClinVar database, including the preferred or alternative disease descriptions with various degrees of association for the variants (Table S1, Supplementary Information). We used rsID from dbSNP^4^ as the identifier to extract the disease terms for the variants from ClinVar, SIFT, Polyphen scores, and the genes associated with the variants from SNPNexus (Chelala et al., 2009; Dayem et al., 2012, 2013). A higher score of 1-SIFT or Polyphen suggests a higher damaging effect of the variants (Chelala et al., 2009; Dayem et al., 2012, 2013). Due to the possibility of multiple variations at some variant loci, we compared various methods to integrate the variant scores for a particular locus, specifically using 1-SIFT or Polyphen scores in combination with the range or the mean score for each variant locus. The gene-variant map with the mean score was applied in the Voronoi-based network analysis to obtain autoimmune associated clusters (Figure 1, Supplementary Information).

The disease terms for subtypes of autoimmune were obtained from the American Autoimmune Related Disease Association (AARDA)^5^, and the autoimmune diseases fact sheet^6^, while some were manually curated from the ClinVar^1,2^ and DISEASES (Pletscher-Frankild et al., 2015) databases. The scores for variants were normalized as the ratio of the sum of the disease-related scores of variants for each chosen gene, divided by the sum of the scores for all variants of the gene. The gene scores were normalized by their maximum scores, which were derived from a single database, i.e., DISEASES (Pletscher-Frankild et al., 2015). We performed the analysis using open source software Python^3^.

We selected 1,383 autoimmune associated genes with Z-scores from the DISEASES database, based on the disease terms of autoimmune and subtypes (Supplementary Information) (Pletscher-Frankild et al., 2015). A total of 1,037 variants with descriptive terms associated with autoimmune from ClinVar^1, 2^ were selected (Supplementary Information). The integrated database contains 85 genes, in which the gene and variant scores reflect the degree of association with autoimmune (Table S2, Supplementary Information).

### Voronoi tessellation and voronoi cell density

Autoimmune related genes, ranked by disease association (Pletscher-Frankild et al., 2015), and the variants for each gene were plotted in the x-y coordinates of the Voronoi diagram. Normally distributed mock data were used to test the Voronoi tessellation and clustering method (Figure S2, Table S4, Supplemental Information). Built-in Voronoi functions in Matlab^7^ were used to create tessellation of data, which returned the indices of the Voronoi cells and vertices.

A Voronoi cell represents an area of influence of the data point it contains, and thus the local density in the proximity of a given point can be determined as the inverse of the cell area. This provides a direct precise measurement of the local density. Clusters were identified based on the neighboring Voronoi cells with densities above a certain threshold. Polyarea, a built-in function in Matlab^7^, was used to calculate the area for each Voronoi cell, except for the cells on the boundary of the map with infinite areas. For Voronoi tessellation with *n* (finite number of) cells, the normalized Voronoi cell density ($\tilde{f}$) was calculated as the ratio of the cell density (inverse of cell area) over the inverse of the average cell area (Ebeling and Wiedenmann, 1993)

(1) $$\begin{array}{l} \tilde{f_{i}}=\frac{f_{i}}{\left(\frac{n}{\sum_{i=1}^{n}1/f_{i}}\right)} \end{array}$$

Parameters were obtained by fitting the Chi-square distribution to 80% of the normalized Voronoi cell density distribution (Ebeling and Wiedenmann, 1993). We obtained the threshold for clustering at the significance level of 90% from the fitted Chi-square distribution (Ramella et al., 2001).

### Identifying clusters of voronoi cells

For the partitioned Voronoi cells, clustering was initiated from a random Voronoi site (data point) *p*, with a normalized Voronoi cell density above the threshold. Then the algorithm visited the neighbors of that random point. Each pair of Voronoi sites was connected by an edge of the Delaunay triangulation. A pair of Voronoi sites was considered as the nearest neighbors if the middle point of the connecting edge was closer to either site from this pair than any other Voronoi sites. Delaunay triangulation *via* the built-in DelaunayTri function in Matlab^7^ was applied to determine the nearest neighbors of the data points. The point *p* and its closest neighbors were included in the neighbor list as a reference for future visit.

A neighbor without prior visit was included in the cluster if its normalized Voronoi cell density was above the threshold. Once visited, the point *p* and its neighbors were added to the visited list and eliminated from the neighbor list. The process was repeated until all the direct or indirect neighbors of the point *p* were exhausted. The entire algorithm was rerun for another random data point without any prior visit until all the data points were exhausted (Edla and Jana, 2011).

### Voronoi-based disease risk

The risk of disease was inferred from the scores of the data points and their corresponding clusters on the gene-variant map. The cluster score was defined as the sum of Voronoi cell densities for all the cells within the cluster. For each gene within a cluster, we considered the product of its cell density and the cluster score, and defined the relative risk as the ratio of the corresponding products for different disease-associated genes. For two data points (corresponding to candidate genes from patients *i* and *j*), the relative risk is thus expressed as

(2) $$\begin{array}{l} Relative\;Risk=\;\frac{f_{i}\sum f_{i}}{f_{j}\sum f_{j}} \end{array}$$

To investigate the contribution of the gene clusters to a disease, the cumulative product of gene scores (*S*_*G*_*i*__ or *S*_*G*_*j*__) and variant scores (*S*_*V*_*i*__ or *S*_*V*_*j*__) for each cluster was calculated, and the relative disease association for the clusters was defined by

(3) $$\begin{array}{l} Relative\;Disease\;Association=\;\frac{\sum S_{G_{i}}S_{V_{i}}}{\sum S_{G_{j}}S_{V_{j}}} \end{array}$$

## Result

### Breast cancer associated genes-variants map

We mapped 15 breast cancer genes with disease association gene Z-scores and the normalized variant scores on the gene-variant map (Figure 1, Figure S1, and Table S3, Supplementary Information). The localization of the genes at the top-right corner of the gene-variant map suggests a high level of association with the disease at both gene and variant levels, implying the relevance to cancer (i.e., high gene scores suggest the relevance to cancer in general) as well as the specificity to breast cancer (i.e., high variant scores). The spatial localization of BRCA1, BRCA2 (Miki et al., 1994; Hofmann and Schlag, 2000), RAD51 (Martin et al., 2007), agrees with the known specificity of these genes to breast cancer, and physical or predicted interactions with each other (Warde-Farley et al., 2010; Zuberi et al., 2013). TP53, the tumor suppressor (Baker et al., 1989; Rivlin et al., 2011), PIK3CA kinase (Karakas et al., 2006), and the epidermal growth factor receptor ErbB-2 and EGFR (Tebbutt et al., 2013), reported in multiple subtypes of cancer, are localized at the bottom right corner of the gene-variant map, suggesting the contribution of these genes to cancer in general with lower specificity to breast cancer compared to BRCA1 and BRCA2 (Figure 1). This map illustrates a spatial segregation of the data points (Figure 1), which may implicate the localization of disease-specific genes. The currently available data pertinent to breast cancer are inadequate for the network analysis of Voronoi tessellation proposed in this study.

### Voronoi based clustering of autoimmune data

**Figure 2:**
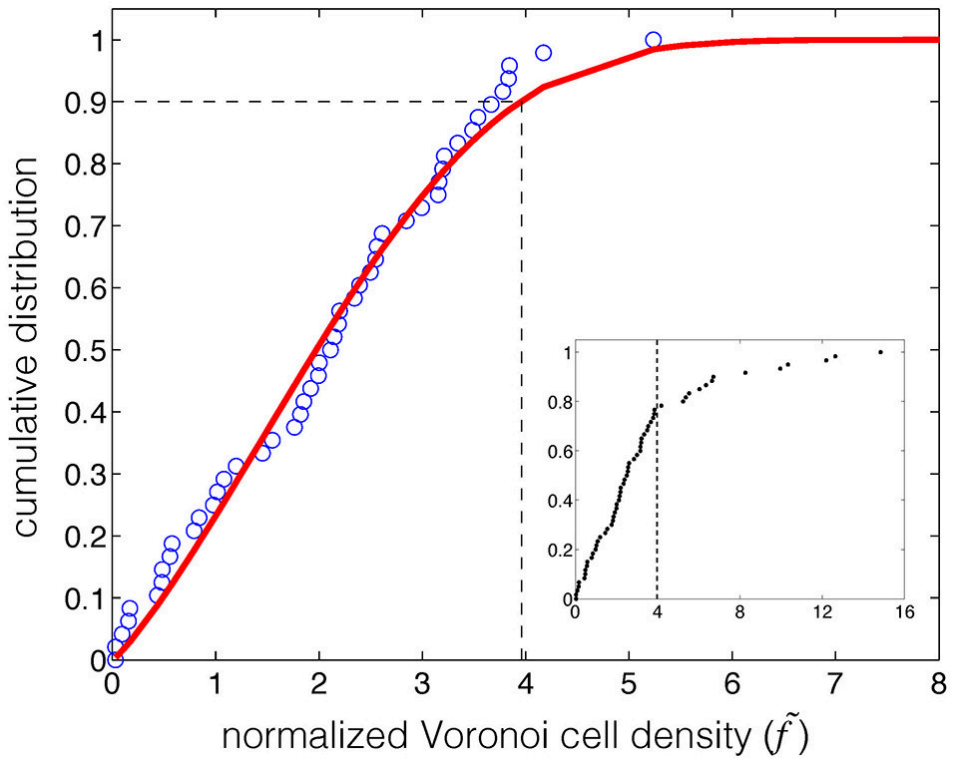
**Identification of the threshold for clustering based on the Chi-square model fitting to the background distribution of the normalized Voronoi cell density of the autoimmune associated gene-variant map**. The red curve represents the fit by the Chi-square distribution to the cumulative distribution of the background (80% of the normalized Voronoi cell density) of autoimmune associated data points (blue circle). The threshold (dashed line) was determined at 3.96, and at the significance level of 90%. The subplot shows the identified threshold (dashed line) on the cumulative distribution of the normalized Voronoi cell density of the entire data points associated with autoimmune.

We mapped 85 autoimmune genes and their associated variants based on the Z-scores and normalized 1-SIFT scores (Table S2, Supplementary Information). We performed Voronoi tessellation and used 80% of the normalized Voronoi cell densities that were lower than or equal to 5.3 as the background distribution (Table S2, Supplementary Information). Voronoi cells with densities above the threshold 3.96 were considered as the candidates for clustering (Figure 2). This threshold was obtained by fitting the Chi-square distribution $\chi_{PDF}\left(cx^{b},\;a\right)$ to the background distribution at the significance level of 90%. The parameters of this distribution were estimated to be *a* = 0.78, *b* = 3, and *c* = 0.04.

**Figure 3:**
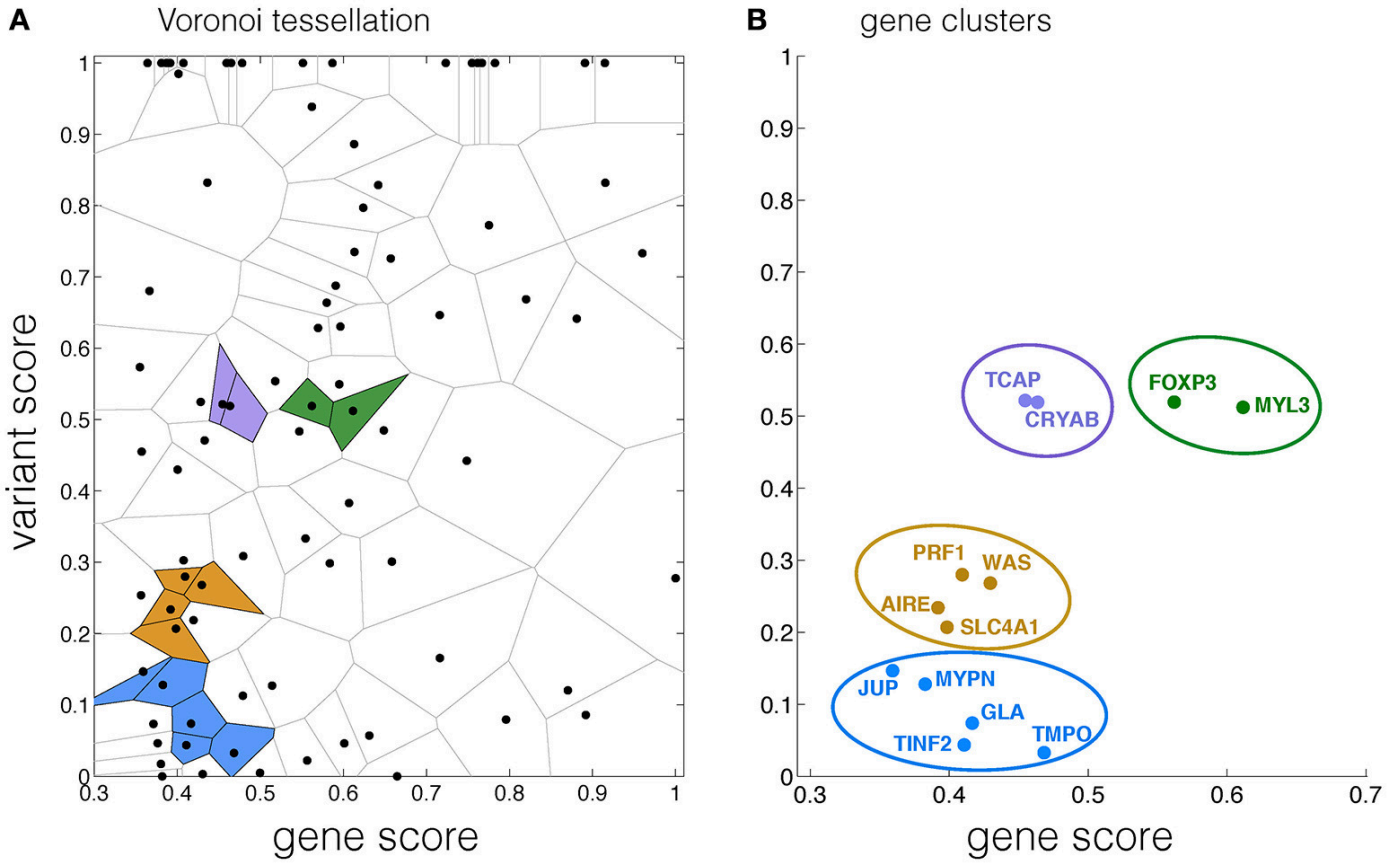
**Autoimmune associated clusters identified by the Voronoi tessellation network analysis**. **(A)** Voronoi tessellation of data points, in which four detected clusters are highlighted in colored cells. **(B)** Genes of data points belonging to the same clusters identified by Voronoi tessellation in **(A)**.

Four clusters associated with autoimmune diseases were detected on the gene-variant map by the Voronoi tessellation network analysis. In cluster 1, the normalized variant and gene (V,G) scores for PRF1, WAS, SLC4A1, AIRE were (0.28,0.41), (0.27,0.43), (0.21,0.4), and (0.23,0.39), respectively. CRYAB, TCAP were found in cluster 2 with normalized (V,G) scores of (0.52,0.46), (0.52,0.45), respectively. The normalized (V,G) scores for FOXP3 and MYL3 in cluster 3 were (0.52,0.56) and (0.51,0.61). In cluster 4, the (V,G) scores for GLA, TMPO, MYPN, JUP, TINF2 were (0.07,0.42), (0.03,0.47), (0.13,0.38), (0.15,0.36), and (0.04,0.41), respectively (Figures 3A,B, Table S2, Supplementary Information). The cluster scores for clusters one to four were 39.41, 16.97, 13.63, and 40.06, respectively (Table S2, Supplementary Information). The localization of cluster 4 on the left bottom corner of the gene-variant map suggests that this cluster contributes less significantly to autoimmune compared with cluster 1, which may in part be explained by its indirect association with autoimmune (Figures 3A,B).

### Risk assessment framework

The multiplication of the normalized Voronoi cell density and its corresponding cluster score provides a disease risk score, from which the risk between patients could be compared (Equation 2). For instance, if a patient with a risk score of 112.71 [identified with mutations in CRYAB, a gene associated with multiple sclerosis (Chauhan et al., 2013)] is compared with another patient who has a risk score of 506.11 [with mutations in TINF2, a gene associated with Idiopathic pulmonary fibrosis (Donahoe et al., 2015)], the relative risk of the former to the latter is 0.22 (Table S2, Supplementary Information). This suggests that the risk of autoimmune diseases would be approximately 5 times higher in the second patient compared with the first patient.

Since the disease association in the gene-variant map is reflected at both gene and variant levels, the contribution of genes identified within a cluster to autoimmune could be calculated as the multiplication of the gene score and the variant score (Equation 3). For instance, the relative risk associated with autoimmune of CRYAB in cluster 2 with the score of *S*_*G*_1__ × *S*_*V*_1__ = 0.24, compared to TINF2 in cluster 4 with the score of *S*_*G*_2__ × *S*_*V*_2__ = 0.02 is 12 (Table S2, Supplementary Information), suggesting a higher relative autoimmune association of CRYAB.

## Discussion

Quantifying the risk of developing a disease based on network analyses of the associated genes, has received much attention over the past two decades. Still, the heterogeneity of complex diseases and synergistic interactions across the network pose enormous challenges for the risk assessment of complex diseases, such as autoimmune. GWAS studies, which mainly focused on common variants, suffer from the shortcomings of missing causal rare variants with low allele frequencies and moderate effects in complex diseases (Bodmer and Bonilla, 2008; Mitchell, 2012). The method of Voronoi tessellation proposed here does not apply any filtering on the frequency of variants available in the databases, and therefore includes both common and rare variants in the score-based clustering. A network analysis that focuses on physical protein-protein interactions or gene co-expressions (Rual et al., 2005; Bettencourt et al., 2016) generally lacks sufficient information on the system-level interactions, which are critical to the understanding of complex diseases.

In this study, we provided a new approach to integrate disease association databases at both gene and variant scales to assess the risk of disease at the system-level, in addition to detecting the associated clusters *via* a Voronoi tessellation analysis. Previous studies on protein-protein network analyses (Ideker and Sharan, 2008; Kuzmanov and Emili, 2013), integrated with GO annotation and biomedical literature mining (Sam et al., 2007), shed lights on the molecular interactions and underlying pathways essential to disease occurrence. GWAS have also been widely used to identify disease-associated variants (Leiserson et al., 2013; van der Sijde et al., 2014). Furthermore, network analyses on protein-protein interactions have been applied with a scoring algorithm to quantitatively analyze the association between diseases (Suratanee and Plaimas, 2015). Therefore, the current surge of interest in quantifying the risk of disease builds on these studies with methods of score-based clustering at the molecular level. Here, we integrated the information available for gene and variant scores with disease association from various repositories and databases to generate a gene-variant map with a spatial gene segregation, in which disease related clusters were identified by a Voronoi tessellation network analysis. For example, the localization of cluster 1 in the center of the map in Figure 3 implies a close association of genes in cluster 1 with autoimmune, corroborating biological pathways (Warde-Farley et al., 2010; Zuberi et al., 2013). This spatial separation of the clusters can provide additional information for comparisons between clusters and their association with the disease, which is critical for the risk assessment.

GeneMANIA suggests that the members of cluster 1 are related to a very tight network, contributing to the Fc receptor signaling pathway and the immune response regulation (Warde-Farley et al., 2010; Zuberi et al., 2013). Cluster 1, localized in the middle of the gene-variant map, implies that its contribution to autoimmune depends on both gene and variant scores (Figures 3A,B). CRYAB was found to be expressed predominantly in multiple sclerosis lesions, suggesting its close association with autoimmune (Ousman et al., 2007). This gene was also identified in cluster 2 containing TCAP (Hayashi et al., 2004), which corroborates previous findings on the co-expression of the two genes (Warde-Farley et al., 2010; Zuberi et al., 2013). GeneMANIA also suggests that cluster 4 belongs to a network functioning in telomere maintenance, recently found to be associated with autoimmune, such as insulin-dependent diabetes mellitus caused by the delayed death of white cells (Jeanclos et al., 1998; Hohensinner et al., 2011). As complex diseases are highly affected by the system-level interactions, our study proposes a network analysis method to identify genes that could potentially contribute to a disease. This presents an important vista for future directions in the field of disease biology network analysis, which currently focuses mainly on direct physical interactions (Bader et al., 2004; Goehler et al., 2004; Gandhi et al., 2006; Chatr-aryamontri et al., 2007; Goh et al., 2007). Furthermore, using the framework presented in this study, the association of genes with different disorders could be modeled by bipartite graph (Goh et al., 2007; Liu et al., 2015) to unravel the contribution of gene clusters to various diseases. While our study aimed to develop a framework that could be used for the disease risk assessment, further investigation is needed to address specificity and sensitivity of the proposed method, given the availability of sufficient amount of patient data (Husmeier, 2003). Nevertheless, our results for clustering autoimmune associated genes presented here show that the method is useful for extracting biologically relevant information.

Our study has several limitations, highlighting the need to improve databases with information of disease association at the variant level. The ambiguous definition of disease terms classified as autoimmune poses a particular challenge for obtaining the associated genes and variants. We obtained such variants from descriptive terms in the xml file (Trait section) of the ClinVar database^1, 2^, in which only a subset of the variants are identified as pathogenic for clinical significance. Further investigations on the association of variants with diseases are needed to provide more accurate variant scores. With improved data quality, our framework could identify clusters associated with various diseases more accurately, which could be used to predict the relative risk based on the proposed network analysis. Depending on the nature of databases and the number of data points, a limitation of our approach, shared with other methods, is identifying the threshold for clustering based on the distribution derived from the selected background data. For example, were a larger amount of data points for genes and variants associated with breast cancer available, our analysis could be used to cluster and segregate data points on the score-based gene-variant map. Furthermore, we have only considered Voronoi cells with finite areas, disregarding possible clustering of data points on the boundary of Voronoi diagram. Despite these limitations, our study highlights the importance of integrating variant scores to the network analysis to identify the contribution of both rare and common variants to disorders. Moreover, the spatial segregation of genes associated with breast cancer from genes related to multiple subtypes of cancer in the Voronoi diagram (Figure 1) suggests that the framework could be used to distinguish subtype-specific genes of complex diseases. These considerations merit further investigation, and our study presents the first step in this direction.

## Author contributions

RD and SM designed the study and contributed to materials and methods. LC participated in the design, collected and analyzed the data, simulated the model and wrote the first draft of the manuscript. GM contributed to interpretation of the results. All authors have contributed to the revision and final draft, and approved its content.

## Funding

This study was in part supported by the Mathematics of Information Technology and Complex Systems (MITACS), and the Natural Sciences and Engineering Research Council of Canada, NSERC Engage grant.

### Conflict of interest statement

GM and RD are members of GeneYouIn Inc., which partially supported this study. LC and SM declare that they have no competing interests.

## Footnotes

^1^http://www.ncbi.nlm.nih.gov/clinvar/

^2^ftp://ftp.ncbi.nlm.nih.gov/pub/clinvar/xml/; last updated on 2015-05-07

^3^*Python Language Reference, version 2.7*. Available online at: http://www.python.org

^4^http://www.ncbi.nlm.nih.gov/SNP/

^5^https://www.aarda.org/autoimmune-information/list-of-diseases/

^6^http://womenshealth.gov/publications/our-publications/fact-sheet/autoimmune-diseases.html

^7^MATLABR2010. The MathWorks, Inc., Natick, Massachusetts, United States.
