# Supplementary material for: Disease Risk Assessment Using a Voronoi-Based Network Analysis of Genes and Variants Scores
Source: Front Genet. 2017 Mar 7;8:29. doi: 10.3389/fgene.2017.00029 (PMC5339255; doi:10.3389/fgene.2017.00029)
Supplement: Supplementary file 5 [file DataSheet1.PDF]

Supplementary Information

Disease Risk Assessment Using a Voronoi-Based Network Analysis of Genes and Variants Scores

Lin Chen[1], Gouri Mukerjee[2], Ruslan Dorfman[2], Seyed M. Moghadas[1]

[1]Agent-Based Modelling Laboratory, York University, Toronto, Canada

[2]GeneYouIn Inc., 16 Ferretti St, Maple ON L6A 0H6

This supplementary information provides details of database integration, analysis of mock data and its Voronoi tessellation, clustering analysis and relevant results.

Integration of databases on genes and variants

Data for the following diseases were obtained from American Autoimmune Related Diseases Association [Reference 46 of the main text]: Wegener's granulomatosis (now termed Granulomatosis with Polyangiitis (GPA)); Inflammatory bowel disease (IBD); Acute Disseminated Encephalomyelitis (ADEM); Antiphospholipid syndrome (APS); Autoimmune inner ear disease (AIED); Autoimmune thrombocytopenic purpura (ATP); Chronic inflammatory demyelinating polyneuropathy (CIDP); Chronic recurrent multifocal osteomyelitis (CRMO); Neuromyelitis optica (Devic's disease); Giant cell arteritis (temporal arteritis); Idiopathic thrombocytopenic purpura (ITP); Juvenile diabetes (Type 1 diabetes); Linear IgA disease (LAD); Lupus (SLE); Lyme disease, chronic; Mixed connective tissue disease (MCTD); Pediatric Autoimmune Neuropsychiatric Disorders Associated with Streptococcus (PANDAS); Pars planitis (peripheral uveitis); Paroxysmal nocturnal hemoglobinuria (PNH); Type I, II, & III

autoimmune polyglandular syndromes; Subacute bacterial endocarditis (SBE); Temporal arteritis/Giant cell arteritis; Thrombocytopenic purpura (TTP); and Undifferentiated connective tissue disease (UCTD).

Inflammatory bowel disease (IBD), Hashimoto's disease, Inflammatory myopathies, Sjögren's (SHOH-grins) syndrome, Systemic lupus erythematosus, were obtained from the autoimmune diseases fact sheet database [Reference 47 of the main text].

Amyloidogenic transthyretin amyloidosis; Hereditary angioedema, type II; Aplastic anemia; Familial dysautonomia, type II; Hereditary pancreatitis; Familial cold urticarial; Spinocerebellar ataxia autosomal recessive with axonal neuropathy; Hereditary motor and sensory neuropathy I; Hereditary motor and sensory neuropathy IB; Familial hypertrophic cardiomyopathy 1; Amyloid polyneuropathy-nephropathy, Iowa type; Progressive familial heart block type 1B; Nodular erythema digital changes; Idiopathic fibrosing alveolitis; Charcot-marie-tooth neuropathy with focal segmental glomerulonephritis; Heinz body hemolytic anemia; Rh-null hemolytic anemia; Progressive myositis ossificans; Mild juvenile diabetes mellitus; Benign chronic pemphigus; Polyglandular autoimmune syndrome, type 1; Pustular psoriasis, generalized; Pyoderma gangrenosum and acne; Spielmeyer Sjogren disease; Sjogren-Larsson Syndrome; Vitiligo-associated multiple autoimmune disease susceptibility were manually curated from ClinVar database [References 39, 40 of the main text].

Lupus erythematosus; asthma; Angioedema; Allergic urticarial; Autoimmune thrombocytopenic purpura; Autoimmune thyroiditis; Acute hemorrhagic leukoencephalitis; Antiphospholipid syndrome; Adult dermatomyositis; Childhood type

dermatomyositis; Transient hypogammaglobulinemia of infancy; Transient hypogammaglobulinemia; Kawasaki disease; Lambert-Eaton myasthenic syndrome; Pure red-cell aplasia; Systemic scleroderma; Demyelinating polyneuropathy were manually curated from DISEASES database [Reference 48 of the main text].

Voronoi tessellation network analysis

Simulated random data points were clustered by Voronoi tessellations, in which the normalized Voronoi cell density of the neighboring cells were above a certain threshold, which was obtained through fitting the Chi-square distribution to the background data (Figure S2 A). Neighboring data points with smaller catchment areas than the threshold were identified in the clusters, which suggest possible interactions between them at the system level, based on their locations on the gene-variant map.

Voronoi tessellation of 500 normally distributed random points were plotted, where the Euclidean space was partitioned into Voronoi cells, each containing one data point as the site (Figure S2 B). We considered less than or equal to 80% of the normalized Voronoi cell density, which is 11.74, as the background distribution [References 28, 30 of the main text] (Figure S2 A, blue circles). A Chi-square distribution (red curve), with parameters a, b, c (as described in the main text), was fitted to the background distribution (Figure S2 A). The fitted parameters were estimated to be 1.58, 3.14, and 0.003 for a, b, and c respectively, with goodness of the fit: R-square of 0.997. The threshold for detecting the clusters of Voronoi tessellation was obtained from the fitted Chi-square cumulative distribution at the 90% significance level. The threshold was selected to be 9.58, so that only cells that have normalized Voronoi cell densities above this threshold were considered as the candidates for clustering. 27 clusters of

Voronoi cells were identified by the clustering algorithm (Figure S2 B, Supplementary Table S4).

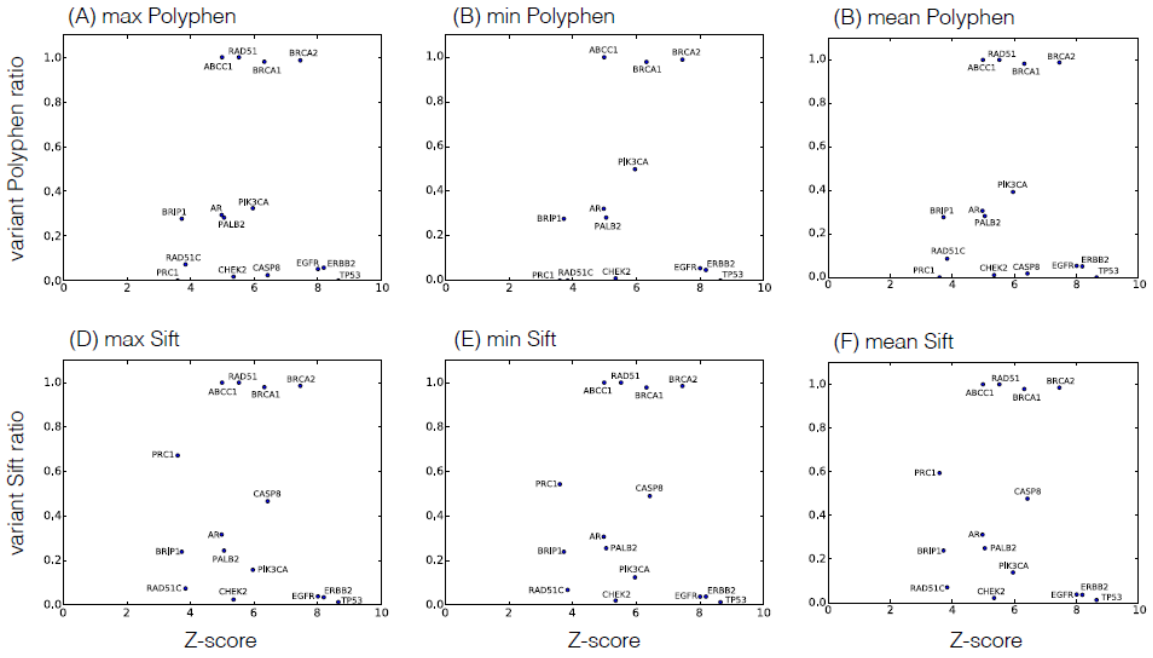

Figure S1. Breast cancer associated gene-variant map, with gene Z-scores (x-axis) and variant scores (y-axis), plotted based on maximum (A, D), minimum (B, E), and mean (C, F) of normalized Polyphen (A, B, C) and 1-SIFT (D, E, F) scores for each variant locus.

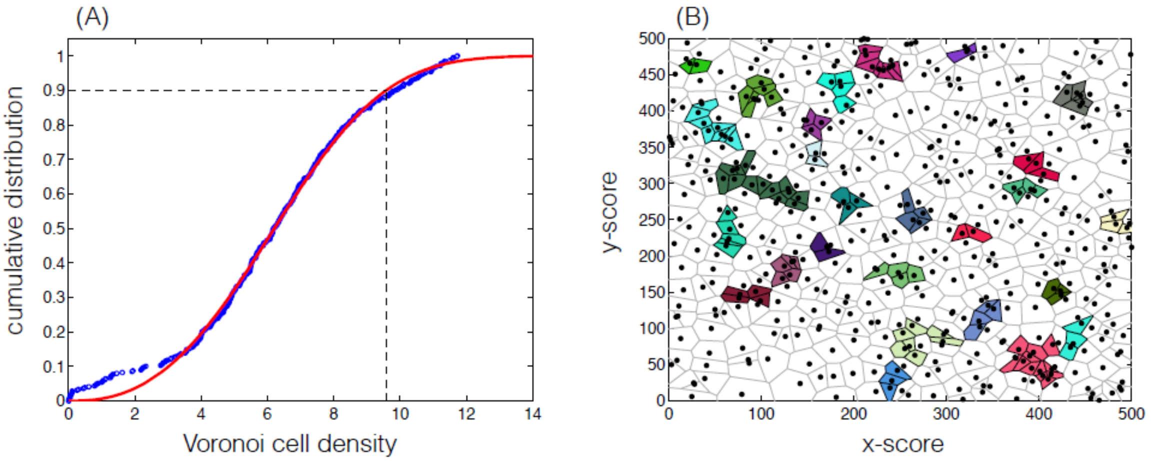

Figure S2. (A) The cumulative distribution of the background data points (blue circles), fitted by the Chi-square distribution (red curve). The threshold (vertical dashed line) was chosen to be 9.58, at the 90% significance level. (B) Voronoi tessellation of the gene-variant map with 27 detected clusters using the threshold identified in (A).
